# Superpixel Segmentation Based on Anisotropic Edge Strength

**DOI:** 10.3390/jimaging5060057

**Published:** 2019-06-05

**Authors:** Gang Wang, Bernard De Baets

**Affiliations:** KERMIT, Department of Data Analysis and Mathematical Modelling, Ghent University, Coupure Links 653, B-9000 Gent, Belgium

**Keywords:** edge strength, first derivative of anisotropic Gaussian kernel, superpixel segmentation, distance measure, graph-based method

## Abstract

Superpixel segmentation can benefit from the use of an appropriate method to measure edge strength. In this paper, we present such a method based on the first derivative of anisotropic Gaussian kernels. The kernels can capture the position, direction, prominence, and scale of the edge to be detected. We incorporate the anisotropic edge strength into the distance measure between neighboring superpixels, thereby improving the performance of an existing graph-based superpixel segmentation method. Experimental results validate the superiority of our method in generating superpixels over the competing methods. It is also illustrated that the proposed superpixel segmentation method can facilitate subsequent saliency detection.

## 1. Introduction

A superpixel is a group of pixels that have similar image properties. Compared with pixels, superpixels embody higher-level features and can sometimes reduce the complexity of subsequent computer vision tasks. With a great many successful applications in object tracking [1], saliency detection [2], stereo matching [3], object detection [4], etc., superpixel segmentation has become a fundamental task in computer vision. Since the superpixel concept was proposed in [5], quite a few superpixel segmentation methods have been developed [6]—most of which can be categorized into partition-based and graph-based methods.

Partition-based superpixel segmentation methods initially partition pixels into different segments that look like grid cells, and then iteratively refine the segments until some convergence criteria are satisfied [7]. A typical example is the method based on simple linear iterative clustering (SLIC method) [8]. The SLIC method first partitions the image into grid cells. Subsequently, in each grid cell, the SLIC method places each cluster center at the location with the lowest local gradient value. Then, all the cluster centers are updated by a *k*-means clustering scheme, in which the distance measure mainly considers the color difference, spatial distance, and compactness constraints. Consequently, the superpixel segmentation result—referred to as the superpixel map—is obtained by the pixel clustering results. The SLIC method can efficiently generate superpixels with good compactness [6]. Nevertheless, the SLIC method makes use of edge strength only in the cluster center initialization step, and as such, the obtained superpixels may not adhere to object contours well, especially when coarse superpixels are desired [9]. Van den Bergh et al. [10] proposed a partition-based method using energy-driven sampling. This method refines the superpixel boundaries according to an energy function that embodies both color and edge information. Although having comparatively low computational cost, this method has difficulty in controlling the desired number of superpixels [7].

Graph-based superpixel segmentation methods group pixels or superpixels into larger superpixels according to graph-based criteria [11]. These methods treat each pixel (or superpixel) as a vertex in a graph and use the distance (i.e., dissimilarity) between two neighboring superpixels as arc weight [6]. Then, the superpixels are obtained by minimizing a cost function defined over the weighted graph. Felzenszwalb and Huttenlocher [12] proposed a method that generates superpixels by determining the minimum spanning tree over the graph. This method uses the Kruskal algorithm to construct the minimum spanning tree and adjusts the criterion according to the degree of variability in neighboring superpixels, thus obeying certain global properties even though greedy decisions are made. Nevertheless, it is difficult for this method to control the desired number of superpixels [7]. Recently, Wei et al. [9] proposed a graph-based method to obtain the so-called superpixel hierarchy (SH method). This method constructs the minimum spanning tree using the Borůvka algorithm, which considers a graph as a forest in which each vertex is initially a tree. These trees grow as the iteration proceeds. For each tree, its aggregate feature value is obtained by aggregating the features (e.g., color) embodied by all the internal vertices. The arc weights are computed as the difference between the aggregate feature values of every two neighboring trees. In each iteration, each tree is merged with the tree that is connected by the arc with the lowest weight. Compared with methods that only use pixel-level features, this method is more robust for segmentation. The SH method benefits greatly from the edge strength, and moreover, it has been confirmed that the segmentation accuracy is dependent on the edge strength measurement methods [9].

With respect to edge strength measurement, which is a classical problem in computer vision, there is a rich body of literature [13]. Existing methods can be roughly classified into three categories: methods based on local kernels, methods based on texture suppression, and methods based on machine learning. Firstly, methods based on kernels identify edges by finding locations where the image intensity shows local changes [14]. Examples are the Sobel method [15], the Canny method [16], and the Laplacian of Gaussian method [17]. However, kernels with small scales are sensitive to noise, while kernels with large scales would blur the image seriously. To address this dilemma, anisotropic Gaussian kernels have been introduced to extract the image gradient map [18,19]. The anisotropic kernels improve the robustness to noise while retaining a good detection of adjacent edges. Besides, in order to handle spatially scaled edges [20], some methods are developed to detect edges using multiscale kernels [21,22]. For example, the method of Bao et al. [23] computes the edge strength by the multiplication of two image gradient maps that are obtained at two different scales. Secondly, methods based on texture suppression are aimed at reducing the textural responses while retaining the edge responses, since textures are not desired in many contour detection tasks. Grigorescu et al. [24] proposed a method based on non-classical receptive field inhibition. Built on the surround inhibition scheme, the method in [25] combines features of orientation, luminance, and luminance contrast, but it incurs heavy computations. Yang et al. [26] proposed a method to compute the spatial sparseness measure. This measure is used to suppress the textural responses in the edge strength map obtained by the so-called color-opponent mechanism. Recently, Akbarinia et al. [27] presented a method that incorporates several biologically inspired models, but this method is relatively time-consuming to execute. Thirdly, methods based on machine learning train edge classifiers using manually annotated samples. Examples are the method using structured forests [28] and the method using deep convolutional neural networks [29]. Such methods have achieved remarkable detection results on publicly available datasets. Nonetheless, their performance might be dependent on the datasets. There is no biological evidence that contour detection requires such a laborious supervised learning progress [27]. In addition, in some specific real-life applications, there is insufficient corresponding ground truth to train a learning model. Unsupervised methods are still required for many tasks [27].

In this paper, aiming at accurate superpixel segmentation, we develop a method of measuring edge strength based on local kernels. The anisotropic edge strength map is obtained by the normalized versions of first derivative of anisotropic Gaussian (FDAG) kernels, which can capture the position, direction, prominence, and scale of the edge to be detected. We also introduce the SH method [9] for superpixel segmentation, in which the distance between neighboring pixels (or superpixels) is determined by both the color information and the edge strength. We modify this work by incorporating the anisotropic edge strength map in the distance measure, and accordingly, a modified superpixel segmentation method is presented.

This paper is organized as follows. Section 2 presents several related works. The method to obtain the anisotropic edge strength is elaborated in Section 3. Subsequently, the obtained anisotropic edge strength is applied to superpixel segmentation in Section 4. Experimental results with accompanying discussions are presented in Section 5, while Section 6 lists our conclusions.

## 2. Related Work

In this section, we explain the concepts of graph-based superpixel segmentation and the anisotropic Gaussian kernel, both of which are quite relevant to our work.

### 2.1. Graph-Based Superpixel Segmentation

Image segmentation refers to the procedure of partitioning an image into segments that have coherent semantic meanings. Therefore, image segmentation methods require either interactions with users or sufficient data to train ad hoc models [30]. By contrast, superpixel segmentation groups pixels that have coherent color or other low-level properties. Therefore, superpixel segmentation usually yields an oversegmented result.

Image segmentation is supposed to be both semantic and hierarchical, because even for a human observer, it is difficult to determine a unique meaningful segmentation of a given image [31]. Illustrations can be found in Section 5. Likewise, the result of superpixel segmentation can also be hierarchical. In most cases, the number of superpixels is manually specified [8]. Among existing superpixel segmentation methods, graph-based methods are more appropriate to generate hierarchical superpixels, since they are usually built on graph theory, which intrinsically provides a hierarchical structure. Moreover, graph-based methods are also efficient to implement.

In mathematics, a weighted graph is a structure consisting of a set of vertices, in which some pairs of vertices are connected by weighted arcs. Particularly, an undirected graph is a graph whose arcs have no orientation. In graph-based superpixel segmentation methods, each pixel in a given image is represented by a vertex, while the distance between two neighboring pixels is represented by an arc weight [12]. Then, the superpixels are obtained by finding the minimum spanning tree of the graph. A spanning tree is an undirected, weighted, and acyclic subgraph. The minimum spanning tree is the spanning tree that has the lowest total arc weight among all the possible spanning trees. Since image segmentation is a procedure of grouping pixels that are meaningfully similar, many graph-based methods segment the image by constructing a minimum spanning tree [32]. In the literature, some superpixel segmentation methods use the Kruskal algorithm [33] and the Borůvka algorithm [9] to construct the minimum spanning tree.

### 2.2. Anisotropic Gaussian Kernels

In the field of computer vision, edges are usually defined as the locations where the image intensity changes sharply. It is straightforward to detect edges by convolutional kernels that can measure the local changes of image intensity. The Canny method is pioneering method which employs the first derivative of an isotropic Gaussian kernel to identify edges [16]. Although having gained popularity, the Canny method is still a monoscale method that can hardly exploit multiscale information. Moreover, when using a small kernel, the Canny method is sensitive to noise. Quite a few methods have been proposed to revise the Canny method. A key development is the use of anisotropic Gaussian kernels to improve the robustness to noise [18,34]. An anisotropic Gaussian kernel is obtained by elongating an isotropic Gaussian kernel [35]. Subsequently, by rotating the kernel, we obtain a directional anisotropic Gaussian kernel:

(1) $$g\left(x;\sigma ,\varphi ,\theta \right)=\frac{1}{2\pi \varphi \sigma^{2}}\exp\left(-\frac{1}{2\sigma^{2}}x^{T}R_{\theta }^{T}\left[\begin{array}{cc} 1 & 0\\ 0 & \varphi^{-2} \end{array}\right]R_{\theta }x\right)\;,$$

where

(2) $$R_{\theta }=\left[\begin{array}{cc} \cos\theta & \sin\theta \\ -\sin\theta & \cos\theta \end{array}\right]$$

is the rotation matrix, $x={\left[x,y\right]}^{T}$ represents the planar coordinates, σ denotes the scale of the kernel, θ stands for the orientation, and φ≥1 is referred to as the anisotropy factor. Accordingly, the first derivative of an anisotropic Gaussian (FDAG) kernel is given by [36]:

(3) $$g^{\prime }\left(x;\sigma ,\varphi ,\theta \right)=-\frac{[\cos\theta ,\sin\theta ]x}{\sigma^{2}}g\left(x;\sigma ,\varphi ,\theta \right)\;.$$

**Figure 1:**
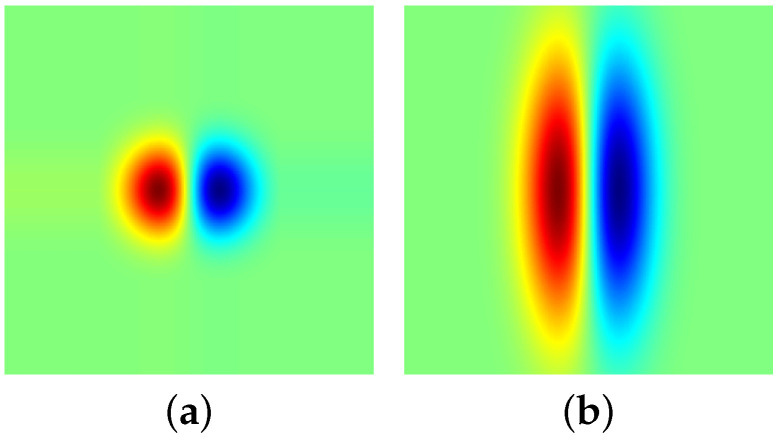
Illustration of two types of the first derivative of Gaussian kernels. (**a**) An isotropic Gaussian kernel; (**b**) A first derivative of anisotropic Gaussian kernel (φ=3).

As an illustration, Figure 1b displays an FDAG kernel, which is visually different from the corresponding isotropic kernel shown in Figure 1a.

Existing FDAG-based methods [19] build a bank of FDAG kernels by setting the anisotropy factor and the direction with possible values. Subsequently, a family of responses is obtained by convolving each kernel with the image. The maximum response among all the kernels is selected as the edge strength. It has been proved that, compared with the kernel in the Canny method, FDAG kernels can significantly improve the robustness to noise [18,19]. Nevertheless, the methods in [18,19] only consider one or two scales in scale space, and therefore underperform in exploiting multiscale information.

## 3. Anisotropic Edge Strength

In this section, we focus on the measurement of edge strength that will be used in the subsequent superpixel segmentation task. The edge strength is obtained by a bank of normalized FDAG kernels. The scale information is identified by FDAG kernels in scale space. The maximum response among all the FDAG kernels and the scale information are combined to yield the anisotropic edge strength. For FDAG kernels, we also design an adaptive anisotropy factor that attenuates the anisotropic stretch effect while retaining a good robustness to noise.

### 3.1. Normalized FDAG Kernels

Many existing methods for edge detection are designed with the assumption that the local changes of image intensity show step appearances. Besides step edges, natural images also contain spatially scaled edges [37]. Therefore, we model the change of image intensity using scaled edges, which can be obtained by the convolutional result of a Gaussian kernel and a step edge as follows:

(4) $$f\left(x;\omega_{0},\theta_{0}\right)=\left(c_{0}\;H\left([\cos\theta_{0},\sin\theta_{0}]x\right)+b_{0}\right)*g\left(x;\omega_{0}\right)\;,$$

where

(5) $$H\left(u\right)=\left\{\begin{array}{cc} 0 & ,\;\mathrm{if}\;u<0\\ 1 & ,\;\mathrm{otherwise} \end{array}\right.$$

denotes the Heaviside step function, and

(6) $$g\left(x;\omega_{0}\right)=\frac{1}{2\pi \omega_{0}^{2}}\exp\left(-\frac{x^{T}x}{2\omega_{0}^{2}}\right)$$

stands for a Gaussian kernel, $\theta_{0}\in [0,\pi [$ denotes the normal direction of the edge, $\omega_{0}\in R_{+}$ represents the edge scale, $c_{0}\in \left[0,1\right]$ is a constant that reflects the extent of the intensity change, and $b_{0}$ $\left(b_{0}+c_{0}\leq 1\right)$ denotes the base level reflecting the background intensity. Since digital images are discrete signals, we always have $\omega_{0}>0$ in practice. In addition, practical edge detection methods usually impose an intrinsic smoothing on the image to suppress noise, and as such, we have $\omega_{0}\geq 1$ in most practical situations.

In the literature, FDAG kernels have been used to obtain the edge strength [18,19,36]. However, conventional FDAG-based methods can hardly exploit multiscale information. To identify the scale of each edge, we normalize the FDAG kernel as follows:

(7) $${\tilde{g}}^{\prime }\left(x;\sigma ,\varphi ,\theta \right)=2\sqrt{\pi }\sigma^{\frac{1}{2}}\cdot g^{\prime }\left(x;\sigma ,\varphi ,\theta \right)\;.$$

Using normalized FDAG kernels, we are able to infer the edge scale in scale space, since the normalized FDAG kernel yields the maximum response at the matched scale (i.e., characteristic scale) of the edge. In addition, the normalization facilitates a quantitative measurement of the intensity change for each edge. We will explain this in more detail below.

It has been proved that, at an appropriate scale, directional FDAG kernels yield the maximum response along the orientation of the edge [19]. That is, when convolving the normalized FDAG kernel in Equation (7) with the edge model in Equation (4) at the scale σ, we have

(8) $$\begin{array}{c} \begin{array}{cc} E_{\mathrm{norm}} & =\max_{\theta }{\left|f\left(x;\omega_{0},\theta_{0}\right)*{\tilde{g}}^{\prime }\left(x;\sigma ,\varphi ,\theta \right)\right|}_{x=0}\\ & ={\left|f\left(x;\omega_{0},\theta_{0}\right)*{\tilde{g}}^{\prime }\left(x;\sigma ,\varphi ,\theta_{0}\right)\right|}_{x=0}\;. \end{array} \end{array}$$

After a few algebraic manipulations, Equation (8) becomes

(9) $$E_{\mathrm{norm}}=\sqrt{2}c_{0}\frac{\sigma^{\frac{1}{2}}}{\sqrt{\omega_{0}^{2}+\sigma^{2}}}\;.$$

In order to identify the scale of the edge, we intend to find the σ at which $E_{\mathrm{norm}}$ yields a maximum response in scale space [38]. For $\sigma \in R_{+}$, it is easy to verify that the second-order derivative of $E_{\mathrm{norm}}$ w.r.t. σ is negative. Therefore, we determine the maximum value of $E_{\mathrm{norm}}$ by computing the first derivative of $E_{\mathrm{norm}}$ w.r.t. σ and setting it to zero. The first derivative of $E_{\mathrm{norm}}$ w.r.t. σ is given by:

(10) $$\frac{\partial E_{\mathrm{norm}}}{\partial \sigma }=\frac{\sqrt{2}}{2}c_{0}\sigma^{-\frac{1}{2}}{\left(\omega_{0}^{2}+\sigma^{2}\right)}^{-\frac{1}{2}}-\sqrt{2}c_{0}\sigma^{\frac{3}{2}}{\left(\omega_{0}^{2}+\sigma^{2}\right)}^{-\frac{3}{2}}.$$

By setting this partial derivative equal to zero, we learn that $E_{\mathrm{norm}}$ obtains its maximum value at the scale

(11) $$\sigma^{*}=\omega_{0}\;.$$

This means that, in scale space, multiscale FDAG kernels yield the maximum response at the characteristic scale of the edge. Moreover, according to Equation (11), at the scale $\sigma^{*}=\omega_{0}$, $E_{\mathrm{norm}}$ obtains its maximum value

(12) $$E_{\mathrm{norm}}^{*}=\frac{c_{0}}{\sqrt{\omega_{0}}}\;,$$

which leads to

(13) $$c_{0}=E_{\mathrm{norm}}^{*}\cdot \sqrt{\omega_{0}}\;.$$

Therefore, having the edge scale $\omega_{0}$ and the maximum response in scale space $E_{\mathrm{norm}}^{*}$, we are able to compute $c_{0}$.

### 3.2. FDAG Kernels with an Adaptive Anisotropy Factor

It has been validated that the non-normalized FDAG kernel obtains higher robustness to noise compared with isotropic Gaussian kernels [18]. Next, we will analyze the noise robustness of the normalized FDAG kernels. We adopt the signal-to-noise ratio (SNR), which is defined as the quotient of the maximum signal response and the standard deviation of the filtered noise [37], to represent the capability of a kernel to suppress noise [39].

Suppose that the scale set of the bank of FDAG kernels is $S=\{\sigma |\sigma_{\min}\leq \sigma \leq \sigma_{\max}\}$ and the image to be processed is corrupted by zero-mean white Gaussian noise ξ(x) with variance $\varepsilon_{0}^{2}$. According to [16,18,37], the intensity of filtered noise is represented by the root-mean-squared response. Thus, at a given scale σ, the intensity of noise filtered by a normalized FDAG kernel is given by [16]:

(14) $$\begin{array}{cc} \varepsilon_{\mathrm{norm}} & =\varepsilon_{0}\sqrt{{\;\int \int \;}_{R^{2}}{\left({\tilde{g}}^{\prime }\left(x;\sigma ,\varphi ,\theta \right)\right)}^{2}\;dx}{|}_{\theta =\theta_{0}}\\ & =\frac{\varepsilon_{0}}{\sigma \sqrt{2\varphi \sigma }}\;. \end{array}$$

It can be seen that $\varepsilon_{\mathrm{norm}}$ has an inverse relationship with the kernel scale σ as well as the anisotropy factor φ. Thus, for a given scale set $S=\{\sigma |\sigma_{\min}\leq \sigma \leq \sigma_{\max}\}$. Kernels that have smaller scales tend to produce larger noise responses, which therefore are more likely to be selected as the final edge strength. Thus, the intensity of noise in the edge strength is mainly determined by the small-scale kernels.

When $\omega_{0}$ is within the interval $\left[\sigma_{\min},\;\sigma_{\max}\right]$, multiscale FDAG kernels yield the maximum response at the scale $\sigma^{*}=\omega_{0}$. According to Equations (12) and (14), the obtained SNR is computed as:

(15) $$\begin{array}{cc} {\mathrm{SNR}}_{\mathrm{norm}}^{*} & =\frac{E_{\mathrm{norm}}^{*}}{\varepsilon_{\mathrm{norm}}}\;{|}_{\sigma =\sigma_{\min}}\\ & =\frac{2c_{0}\sigma_{\min}^{2}\sqrt{\varphi }}{\varepsilon_{0}\sqrt{2\omega_{0}\sigma_{\min}}}\;. \end{array}$$

Therefore, the proposed normalized FDAG kernel (φ>1) obtains a higher SNR than the isotropic kernel (φ=1). Also, when we use FDAG kernels to detect an edge in a noisy image, the SNR is mainly determined by φ and $\sigma_{\min}$. Thus, we learn that it is not necessary to apply anisotropy factors in kernels at all the scales, especially in kernels at large scales, since the latter can ensure a good robustness to noise even if the anisotropy factor is absent.

The use of FDAG kernels incurs an anisotropy stretch effect [18], because the blurring extent w.r.t. *x* is determined by σ, while the blurring extent w.r.t. *y* is determined by φσ. To address this problem, we embed an adaptive anisotropy factor in FDAG kernels.

Given a scale set $S=\{\sigma |\sigma_{\min}\leq \sigma \leq \sigma_{\max}\}$, we obtain an adaptive anisotropy factor as follows:

(16) $$\varphi \left(\sigma \right)=\left\{\begin{array}{cc} \frac{\sigma_{\mathrm{con}}^{2}}{\sigma^{2}} & ,\;\mathrm{if}\;\sigma_{\min}\leq \sigma \leq \sigma_{\mathrm{con}},\\ 1 & ,\;\mathrm{otherwise}, \end{array}\right.$$

where $\sigma_{\mathrm{con}}\in \left[\sigma_{\min},\;\sigma_{\max}\right]$ stands for the robustness control scale. On the one hand, when we use kernels at $\sigma \leq \sigma_{\mathrm{con}}$, we introduce a large anisotropy factor to improve the robustness to noise. On the other hand, when we use a kernel at $\sigma >\sigma_{\mathrm{con}}$, the anisotropy factor is set as φ=1 because a large σ is already able to guarantee a good robustness to noise.

Eventually, by substituting Equation (16) into Equations (3) and (7), we get an FDAG kernel that incorporates an adaptive anisotropy factor.

### 3.3. Discrete Version of FDAG Kernels

**Figure 2:**
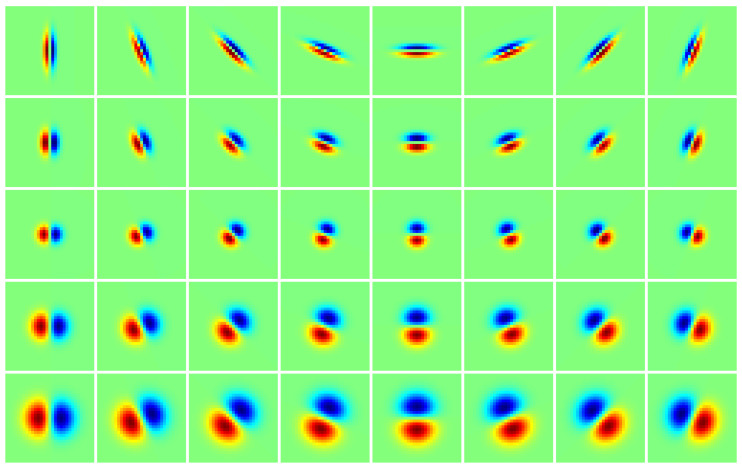
Examples of discrete normalized first derivative of anisotropic Gaussian (FDAG) kernels. The control scale is set as $\sigma_{\mathrm{con}}=2$. **Top row:** Kernels with σ=1 and φ=4. **Second row:** Kernels with σ=1.5 and φ=1.78. **Third row:** Kernels with σ=2 and φ=1. **Fourth row:** Kernels with σ=3 and φ=1. **Bottom row:** Kernels with σ=4 and φ=1. The intensity range of each patch has been adjusted for better display.

In order to accommodate the normalized FDAG kernels to digital image processing, we obtain discrete versions of FDAG kernels by sampling the formulae in Equations (1), (3), and (7) in the 2D integer coordinate $Z^{2}$ as follows:

(17) $$\begin{array}{cc} g(m;\sigma_{i},\varphi_{i},\theta_{j}) & =\frac{1}{2\pi \varphi_{i}\sigma_{i}^{2}}\exp\left(-\frac{1}{2\sigma_{i}^{2}}m^{T}R_{\theta_{j}}^{T}\left[\begin{array}{cc} 1 & 0\\ 0 & \varphi_{i}^{-2} \end{array}\right]R_{\theta_{j}}m\right),\\ {\tilde{g}}^{\prime }\left(m;\sigma_{i},\varphi_{i},\theta_{j}\right) & =-2\sqrt{\pi }\sigma_{i}^{\frac{1}{2}}\cdot \frac{[\cos\theta_{j},\sin\theta_{j}]m}{\sigma_{i}^{2}}g\left(m;\sigma_{i},\varphi_{i},\theta_{j}\right)\;, \end{array}$$

where $m={\left[m_{x},m_{y}\right]}^{T}\in Z^{2}$ represents the digital image coordinates, $\sigma_{i}$ stands for the scale taking values from a scale set S, and

(18) $$R_{\theta_{j}}=\left[\begin{array}{cc} \cos\theta_{j} & \sin\theta_{j}\\ -\sin\theta_{j} & \cos\theta_{j} \end{array}\right]$$

represents the rotation matrix. $\theta_{j}$ denotes the direction taking values from a direction set D, while

(19) $$\varphi_{i}=\max\;(\frac{\sigma_{\mathrm{con}}^{2}}{\sigma_{i}^{2}},\;1)$$

is used to compute the adaptive anisotropy factor. For illustration, Figure 2 displays some samples of normalized FDAG kernels.

### 3.4. Anisotropic Edge Strength

Having discrete versions of FDAG kernels, we are able to obtain the anisotropic edge strength map. To process color images that consist of three channels, we convolve the bank of FDAG kernels with each channel as follows:

(20) $$E^{q}\left(m;\sigma_{i},\varphi_{i},\theta_{j}\right)=\left|I^{q}\left(m\right)*{\tilde{g}}^{\prime }\left(m;\sigma_{i},\varphi_{i},\theta_{j}\right)\right|\;,$$

where $I^{q}$ (q∈{1,2,3}) denotes the *q*-th channel of a given color image *I*.

Subsequently, at each position, the maximum response among all directions, scales, and channels is selected as follows [40,41]:

(21) $$E\left(m\right)=\max_{q}\;\max_{\sigma_{i}\in S}\;\max_{\theta_{j}\in D}\;E^{q}\left(m;\sigma_{i},\varphi_{i},\theta_{j}\right)\;.$$

On the one hand, as elaborated earlier, the response of the bank of FDAG kernels reaches the maximum value at the characteristic scale in scale space. Therefore, for each edge, the scale is estimated by maximizing the response in scale space:

(22) $$S\left(m\right)=\underset{\sigma_{i}\in S}{arg max}\;\max_{q}\;\max_{\theta_{j}\in D}\;E^{q}\left(m;\sigma_{i},\varphi_{i},\theta_{j}\right)\;.$$

On the other hand, since FDAG kernels yield the maximum response along the direction of the edge [16,18], we obtain the edge direction map by maximizing the response as follows:

(23) $$\Theta \left(m\right)=\underset{\theta_{j}\in D}{arg max}\;\max_{q}\;\max_{\sigma_{i}\in S}\;E^{q}\left(m;\sigma_{i},\varphi_{i},\theta_{j}\right)\;.$$

According to Equation (13), we should compute the final response using both the maximum response E and the scale map *S* at all positions. Actually, in edge (or contour) detection, only the positions on the centerlines of edges (or contours) may appear in the detection result. Thus, for the sake of computational efficiency, we only consider the candidates of the edge centerlines. The candidate positions of the edge centerlines can be identified by the technique of nonmaxima suppression (NMS) [42] on the anisotropic edge strength map. Denoting the result of the NMS technique as $E_{\mathrm{nms}}$, we obtain the anisotropic edge strength map as follows:

(24) $$I_{e}\left(m\right)=\max\left(E\left(m\right),\;E_{\mathrm{nms}}\left(m\right)\cdot \sqrt{S(m)}\right)\;.$$

**Figure 3:**
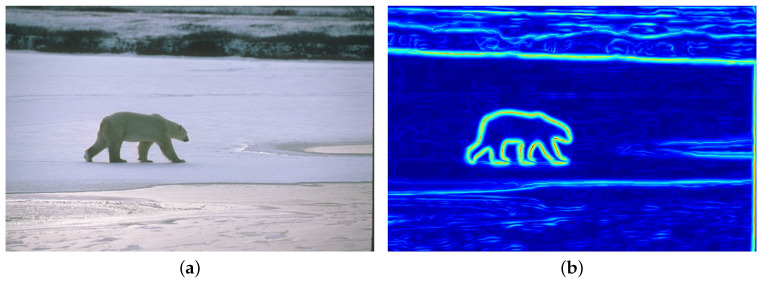
Illustration of the anisotropic edge strength map (**b**) obtained on the original image (**a**).

As an illustration, Figure 3b displays the anisotropic edge strength map obtained on Figure 3a.

## 4. Superpixel Segmentation Based on Anisotropic Edge Strength

In this section, we introduce the graph-based SH method [9] for superpixel segmentation. The SH method uses a graph to represent the image, and constructs a minimum spanning tree using the Borůvka algorithm to obtain the superpixel map. In order to improve its performance in segmentation accuracy, we incorporate the anisotropic edge strength obtained in Section 3 into the distance measure between neighboring superpixels.

### 4.1. Preliminary Superpixel Map Obtained by Pixel Grouping

In graph-based image processing, given an image *I*, each pixel I(m) is represented by a vertex $v_{i}\in V$ and the distance between two neighboring pixels, $v_{i}$ and $v_{j}$, is represented by an arc weight $d(v_{i},v_{j})\in D$. Then, V and D form an undirected graph G=(V,D). To construct a minimum spanning tree, initially, for each vertex, the Borůvka algorithm finds its nearest neighbor in terms of the Euclidean distance in color space, and then groups them into a single tree. Subsequently, for each tree, the Borůvka algorithm finds its nearest neighbor. Denoting a pair of neighboring trees by $T_{1}$ and $T_{2}$, we compute the distance between them as follows:

(25) $$D\left(T_{1},T_{2}\right)=\min_{v_{i}\in T_{1},\;v_{j}\in T_{2}}d\left(v_{i},v_{j}\right)\;.$$

Essentially, $D(T_{1},T_{2})$ is a pixel-level distance measure, and as such, it is reasonable when the number of vertices in each tree is small. However, as the trees grow as the iteration proceeds, $D(T_{1},T_{2})$ can hardly reflect the dissimilarity between a pair of neighboring trees. Therefore, the pixel grouping procedure is carried out for τ iterations, where τ∈Z should be set to a small value. According to the parameter settings in [9], τ is set as 4.

### 4.2. Superpixel Segmentation

We obtained the preliminary superpixel map by a pixel grouping procedure, in which each superpixel is represented by a tree in a graph. Nevertheless, as mentioned earlier, the distance measure defined in Equation (25) is inappropriate for use in segmenting superpixels at higher hierarchy levels, since it only makes use of pixel-level features. As the superpixel grows, the pixel-level features are sensitive to outliers such as noisy pixels. Therefore, in the following iterations, the distance between a pair of neighboring trees is defined as follows:

(26) $$D\left(T_{1},T_{2}\right)=D_{e}\left(T_{1},T_{2}\right)\cdot D_{c}\left(T_{1},T_{2}\right)\;,$$

where $D_{e}$ represents the mean edge strength of the pixels that are situated on the shared border between $T_{1}$ and $T_{2}$. In the superpixel segmentation method presented in [9], the edge strength is obtained by the learning-based structured forest method [28]. In this paper, we use the FDAG-based anisotropic edge strength obtained in Equation (24) to compute $D_{e}\left(T_{1},T_{2}\right)$.

In addition, in Equation (26), $D_{c}$ denotes the Chi-Squared histogram distance [43] between $T_{1}$ and $T_{2}$, which is computed as [30]:

(27) $$\begin{array}{cc} D_{c}\left(T_{1},T_{2}\right) & =\chi^{2}\left(h_{T_{1}},h_{T_{2}}\right)\\ & =\frac{1}{2}\sum_{i}^{N_{\mathrm{bin}}}\frac{{\left(h_{T_{1}}\left(i\right)\;-\;h_{T_{2}}\left(i\right)\right)}^{2}}{h_{T_{1}}\left(i\right)\;+\;h_{T_{2}}\left(i\right)}\;, \end{array}$$

where $h_{T_{1}}$ and $h_{T_{2}}$ are the color histograms of $T_{1}$ and $T_{2}$, respectively, *i* denotes the index of bins, and $N_{\mathrm{bin}}$ stands for the number of bins in each histogram. According to the parameter settings in [9], $N_{\mathrm{bin}}$ is set to 20.

According to the distance measure in Equation (26), for each tree, the Borůvka algorithm finds its nearest tree and groups them into a single tree. As the iteration continues, superpixel maps at higher hierarchy levels can be obtained.

## 5. Experimental Validation

We presented a method to compute the FDAG-based anisotropic edge strength, which is summarized in Algorithm 1. Building on this, we also presented the SH-based superpixel segmentation method, which incorporates the FDAG-based edge strength (SH+FDAG method). In order to test whether or not the SH+FDAG method works well, we used our method to obtain superpixels on three publicly available datasets, including the Berkeley segmentation dataset and benchmarks 500 (BSDS500; https://www2.eecs.berkeley.edu/Research/Projects/CS/vision/grouping) [30], the systematic benchmarking for aerial image segmentation dataset (SBAIS; http://jiangyeyuan.com/ASD) [44], and the neuronal structures in electron microscopy stacks dataset (NSEMS; http://brainiac2.mit.edu/isbi_challenge/home) [45]. In addition, in order to explore the impact of different kinds of edge strength on the segmentation accuracy, we also tested the SH methods that incorporate edge strength obtained by the structured forest edge (SH+SFE) method [28], the sparseness-constrained color-opponency (SH+SCO) method [26], the automated anisotropic Gaussian kernel (SH+AAGK) method [19], and the surrounded-modulation edge detection (SH+SED) method [27]. Furthermore, we also selected the widely used SLIC method [8] for comparison.
**Algorithm 1** The proposed method to compute the anisotropic edge strength.**Require:** Image *I*, scale set S, direction set D, control scale $\sigma_{\mathrm{con}}$**Ensure:** Anisotropic edge strength $I_{e}$ 1:**for** each $I^{q}\in I$ **do** 2:    **for** each $\sigma_{i}\in S$ **do** 3:        $\varphi_{i}\leftarrow \max\;\left(\frac{\sigma_{\mathrm{con}}^{2}}{\sigma_{i}^{2}},\;1\right)$ 4:        **for** each $\theta_{j}\in D$ **do** 5:           $E^{q}\left(m;\sigma_{i},\varphi_{i},\theta_{j}\right)\leftarrow \left|I^{q}\left(m\right)*{\tilde{g}}^{\prime }\left(m;\sigma_{i},\varphi_{i},\theta_{j}\right)\right|$ 6:        **end for** 7:    **end for** 8:**end for** 9:$E\left(m\right)\leftarrow \max_{q}\max_{\sigma_{i}\in S}\;\max_{\theta_{j}\in D}\;\;E^{q}\left(m;\sigma_{i},\varphi_{i},\theta_{j}\right)$10:$S\left(m\right)\leftarrow \underset{\sigma_{i}\in S}{arg max}\;\max_{q}\max_{\theta_{j}\in D}\;E^{q}\left(m;\sigma_{i},\varphi_{i},\theta_{j}\right)$11:$\Theta \left(m\right)\leftarrow \underset{\theta_{j}\in D}{arg max}\;\max_{q}\max_{\sigma_{i}\in S}\;E^{q}\left(m;\sigma_{i},\varphi_{i},\theta_{j}\right)$12:$E_{\mathrm{nms}}\left(m\right)\leftarrow$ NMS using E(m) and Θ(m)13:$I_{e}\left(m\right)\leftarrow \max\left(E\left(m\right),\;E_{\mathrm{nms}}\left(m\right)\cdot \sqrt{S(m)}\right)$

### 5.1. Evaluation Metrics

In order to evaluate the segmentation accuracy, we adopted two widely used evaluation measures: the Achievable Segmentation Accuracy (ASA) and the undersegmentation error (UE).

The ASA reflects the fraction of ground truth (GT) segments that are correctly labelled by superpixels. When compared with the GT, a correctly segmented superpixel is supposed to be totally contained in a GT segment. Otherwise, the superpixel overlaps with more than one GT segment. Then, we used the label value of the GT segment that has the largest overlapping region with this superpixel to label all the pixels within this superpixel. Consequently, a map *L* consisting of such labels was obtained. Comparing *L* with the GT, we computed the fraction of the correct labels in *L* as the ASA. That is, given a superpixel map $I_{\mathrm{sp}}$ and the corresponding GT $I_{\mathrm{gt}}$, the ASA is computed as [9]:

(28) $$\mathrm{ASA}=\frac{1}{\sum_{G_{j}\in I_{\mathrm{gt}}}\left|G_{j}\right|}\sum_{S_{i}\in I_{\mathrm{sp}}}\max_{G_{j}\in I_{\mathrm{gt}}}\left|S_{i}\cap G_{j}\right|\;,$$

where $S_{i}$ and $G_{j}$ denote the segments in $I_{\mathrm{sp}}$ and $I_{\mathrm{gt}}$, respectively, and |·| stands for the number of pixels. For a superpixel segmentation method, a higher ASA value is preferred.

The UE reflects the *leakage* of superpixels with respect to the corresponding GT [6]. Comparing the superpixels with the GT, we affiliated each superpixel with the GT segment that had the largest mutually overlapping region. Then, within each superpixel, the pixels that incorrectly matched the GT segment were considered as *leakage*. Given a superpixel map $I_{\mathrm{sp}}$ and the corresponding GT $I_{\mathrm{gt}}$, the UE is computed as follows [6]:

(29) $$\mathrm{UE}=\frac{1}{\sum_{G_{j}\in I_{\mathrm{gt}}}\left|G_{j}\right|}\sum_{G_{j}\in I_{\mathrm{gt}}}\sum_{S_{i}\cap G_{j}\neq \emptyset }\min\left\{\left|S_{i}\cap G_{j}\right|,\;\left|S_{i}\setminus G_{j}\right|\right\}\;,$$

where $S_{i}$ and $G_{j}$ denote the segments in $I_{\mathrm{sp}}$ and $I_{\mathrm{gt}}$, respectively, and ∖ stands for the set difference operation. For superpixel segmentation, a lower UE is preferred.

### 5.2. Parameter Settings

As mentioned earlier, we selected several methods, including the SLIC, SH+SFE, SH+SCO, SH+AAGK, and SH+SED methods, as the competing methods. To make the evaluation results reproducible, we list the parameter settings of each method below:

- SLIC: According to the original implementation, the compactness was set to 10 [8].
- SH+SFE: The compactness parameter was set as 0.53. The SFE method, in which the number of trees was set as 4, was trained on the BSDS500 dataset [28].
- SH+SCO: The compactness parameter was set as 0.53. According to the parameter setting reported in [26], the size of the receptive field was set as 1.1. The number of orientations was set as 8. The connection weights from cones to retinal ganglion cells were set as 0.7 and −0.7, respectively. The size of the local window for sparseness measure was set as 11.
- SH+AAGK: The compactness parameter was set as 0.53. According to [19], the number of kernel orientations was set as 8, and accordingly, the scale and the anisotropy factor were computed as $\sqrt{10}$ and $\sqrt{5}$, respectively.
- SH+SED: The compactness parameter was set as 0.53. Adopting the implementation of SED in [27], the size of the receptive field in the lateral geniculate nucleus layer and in the primary visual cortex was set as 0.5 and 1.5, respectively. The number of directions was set as 12.
- SH+FDAG: The compactness parameter was set as 0.53. The number of directions was set as 8. The scale set was configured as S={1.0,1.1,1.2,…,1.5}, while the control scale was set as 1.2.

### 5.3. Evaluation on the BSDS500 Dataset

**Figure 4:**
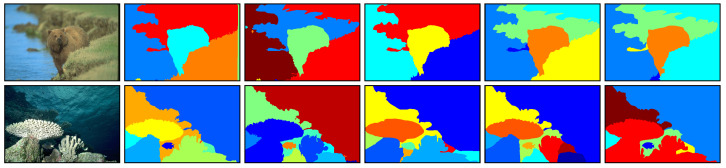
Sample images as well as their multiple ground truth (GT) segmentation maps taken from the Berkeley segmentation dataset and benchmarks 500 (BSDS500) dataset. **Left column:** Original images. **Second to sixth columns:** GT segmentation maps labelled by different annotators for each original image.

The BSDS500 dataset contains 200 test images and represents the diversity of natural images. The resolution of each image is either 481×321×3 or 321×481×3. The corresponding GT segmentation maps of each test image are labelled by different annotators [30]. We illustrate two sample images as well as their multiple GT segmentation maps in Figure 4. For each image, when comparing a superpixel map with the multiple GT segmentation maps, we obtained multiple ASA and UE values. The best values were retained as the evaluation results.

**Figure 5:**
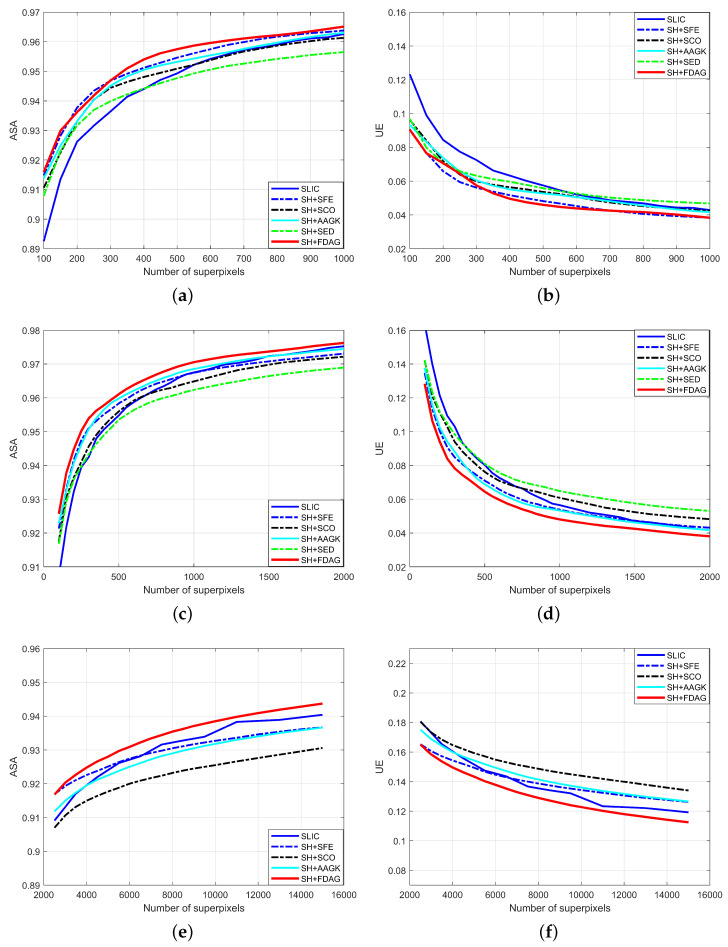
Evaluation results in terms of the average Achievable Segmentation Accuracy (ASA) and average undersegmentation error (UE). (**a**,**b**): Results obtained on the BSDS500 dataset. (**c**,**d**): Results obtained on the systematic benchmarking for aerial image segmentation (SBAIS) dataset. (**e**,**f**): Results obtained on the neuronal structures in electron microscopy stacks (NSEMS) dataset.

For each method, the curves of the average ASA and average UE values over all 200 images with respect to different numbers of superpixels are displayed in Figure 5a,b. Among the SH-based methods, the SH+FDAG and SH+SFE methods obtained a competitive performance, achieving higher average ASA values and lower average UE values than other methods. This indicates that the edge strength maps obtained by the FDAG and SFE method are more appropriate for superpixel segmentation. By comparison, the SH+SCO and SH+AAGK methods obtained slightly worse performances, while the SH+SED method obtained a limited performance. The main reason is that the SED method mainly focuses on texture suppression. The edge strength map yielded by the SED method was usually fragmented. Such edge strength will inevitably lead to errors when used in the SH method for superpixel segmentation. Moreover, compared with SH-based methods, the SLIC method obtained a competitive performance when the number of superpixels was large, but underperformed when coarse superpixels were desired. This is mainly because the SLIC method is a partition-based method. It takes the edge strength into consideration only in the initialization step.

**Figure 6:**
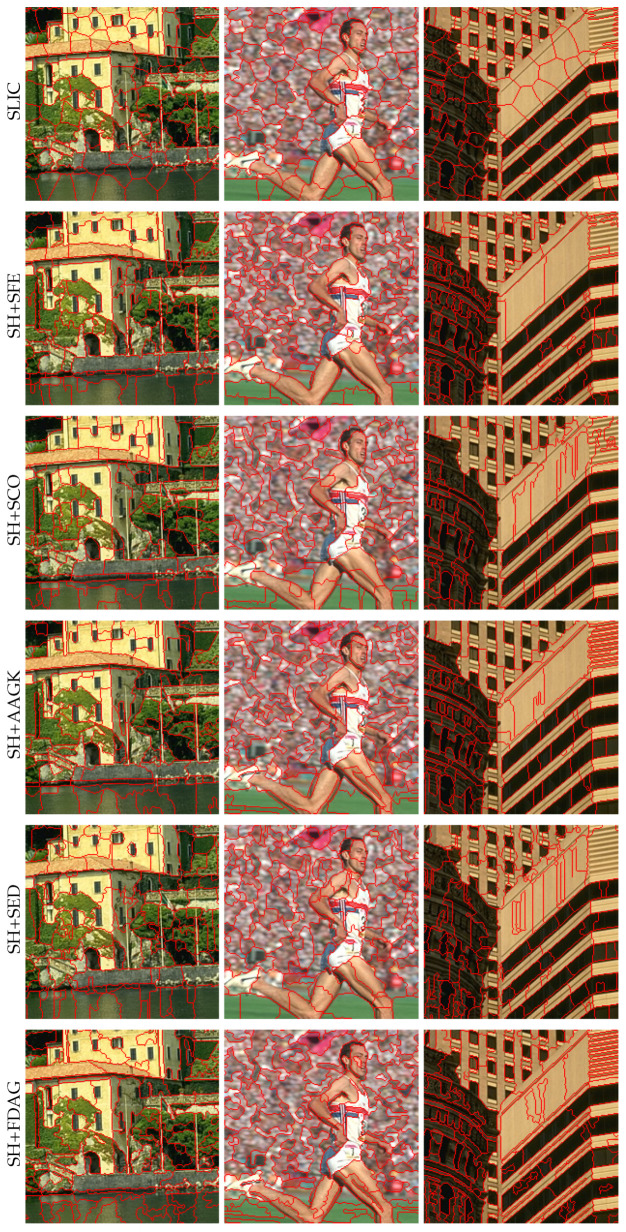
Superpixel maps yielded by different methods on sample images taken from the BSDS500 dataset. The number of superpixels in each full superpixel map was set to 400. For a better visualization, zoomed-in versions are displayed. SH: superpixel hierarchy; SLIC: simple linear iterative clustering; SH+AAGK: SH method incorporating edge strength obtained by the automated anisotropic Gaussian kernel; SH+FDAG: SH method incorporating the FDAG-based edge strength; SH+SCO: SH method with edge strength obtained by sparseness-constrained color-opponency; SH+SED: SH method incorporating edge strength obtained by surrounded-modulation edge detection; SH+SFE: SH method incorporating edge strength obtained by the structured forest edge method.

Besides the quantitative evaluation results, we also illustrate sample superpixel maps yielded by different methods in Figure 6. It can be seen that, compared with the SLIC method, the SH-based methods—especially the SH+FDAG and SH+SFE methods—adhered better to the object contours.

**Figure 7:**
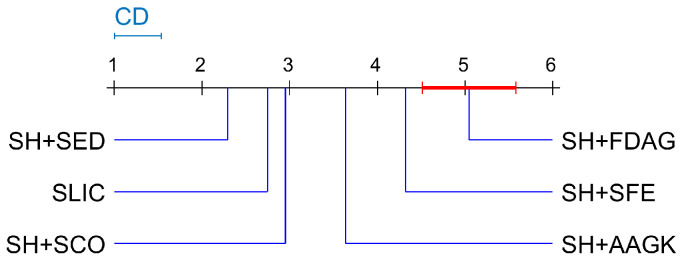
Visualization of the post hoc Nemenyi test performed on the average ASA evaluation results when the number of superpixels was 500, obtained on the BSDS500 dataset.

In order to see whether or not the quantitative evaluation results of our method were significantly different from those of other methods, we selected the cases corresponding to 100, 300, 500, 700, and 900 superpixels, respectively. For each case, we applied the non-parametric Friedman test, which is similar to repeated-measures ANOVA, to test the null hypothesis that all the methods obtain the same performance in terms of the average ASA and the average UE, respectively. The *p*-value results of the Friedman test are presented in Table 1. As indicated by these results, the null hypothesis was rejected in each case. That is, the average ASA and average UE values obtained by different methods had significant differences in each case. Then, in the post-hoc tests, we applied the Nemenyi test, selecting our method as the control method. In the Nemenyi test, the critical distance (CD) is:

(30) $$\mathrm{CD}=q_{\alpha }\times \sqrt{\frac{n_{m}\left(n_{m}+1\right)}{6n_{s}}}\;,$$

where $n_{m}$ and $n_{s}$ denote the number of methods and the number of image samples, respectively. In the experiment performed on the BSDS500 dataset, the CD was 0.5332. Compared with the control method, a method was considered significantly different if its average rank differed by at least one CD from the average rank of the control method. For instance, Figure 7 visualizes the significant differences among the average ASA values of different methods when the number of superpixels was 500. As can be seen, the average rank of each competing method was outside of the marked area, so each competing method was considered as significantly different from the control method. The average rank of each method in each selected case is reported in Table 2 and Table 3. It can be seen that in most cases, the SLIC, SH+SCO, SH+AAGK, and SH+SED methods were significantly different from the SH+FDAG method, while the SH+SFE and SH+FDAG methods achieved a similar performance.

### 5.4. Evaluation on the SBAIS Dataset

**Figure 8:**
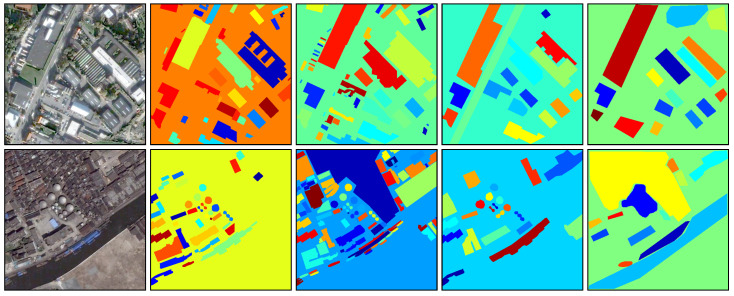
Sample images as well as their multiple GT segmentation maps taken from the SBAIS dataset. **Left column:** Original images. **Second to fifth columns:** GT segmentation maps labelled by different annotators for each original image.

There are 80 aerial images in the SBAIS dataset, each of which has a resolution of 512×512×3. For each image, there are four manually labelled GT segmentation maps. Two sample images as well as the GT segmentation maps are displayed in Figure 8. For each aerial image, we obtained four ASA values and four UE values when comparing a superpixel map with the multiple GT segmentation maps. The best ASA and UE values for each image were retained as the evaluation results.

For each method, the curves of average ASA and average UE values over all the 80 images with respect to different numbers of superpixels are shown in Figure 5c,d. It can be seen that our method performed the best among all the methods, obtaining higher average ASA values and lower average UE values than the competing methods. Note that the SH+SFE method did not obtain the best performance in terms of the average ASA and average UE, which is different from the results obtained on the BSDS500 dataset. This is mainly because the edge detection model in the learning-based SFE method was trained on the BSDS500 dataset, which consists of natural images. As a result, the SH+SFE method underperformed on the aerial images from the SBAIS dataset. In addition, the SH+SED method, the edge strength maps of which are not smooth, still performed the worst among all the SH-based methods. Compared with the SH-based methods, the SLIC method obtained a competitive performance when the number of superpixels was large (i.e., when the hierarchy level was low), but underperformed when the number of superpixels was small. This is consistent with the experimental results obtained on the BSDS500 dataset. As mentioned earlier, the main reason is that the SLIC method is a partition-based method. It does not consider the edge strength during the process of pixel clustering.

**Figure 9:**
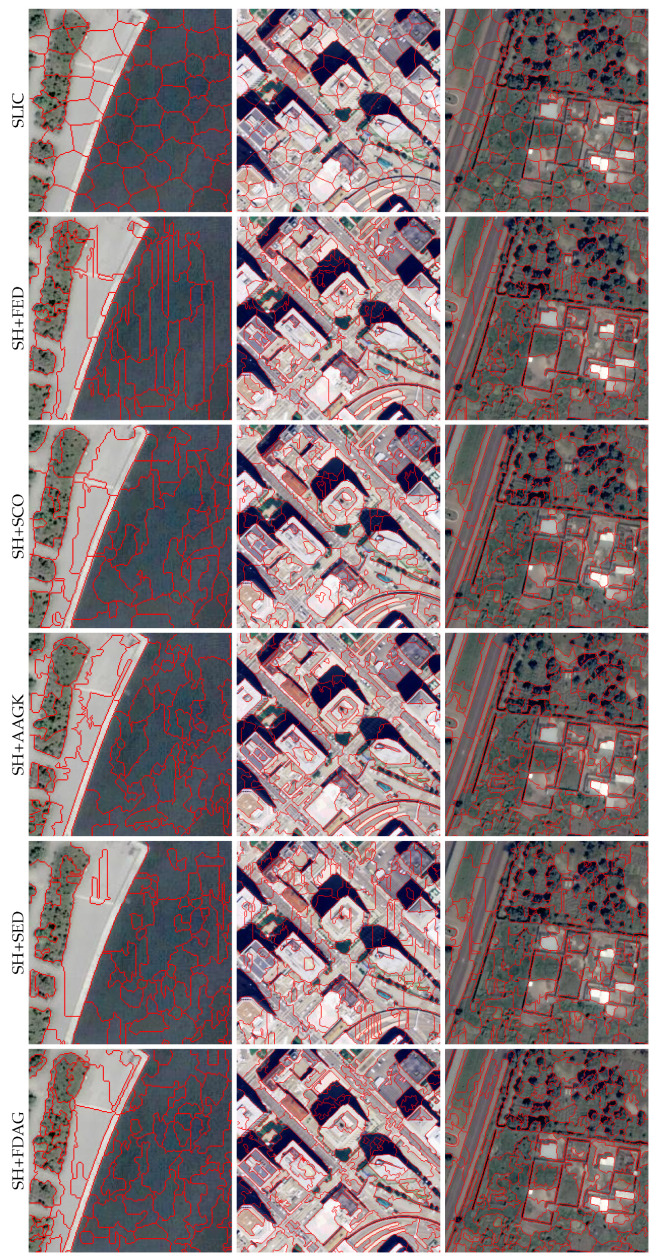
Superpixel maps yielded by different methods on sample images taken from the SBAIS dataset. The number of superpixels in each full superpixel map was set as 500. For a better visualization, zoomed-in versions are displayed.

The superpixel maps yielded by different methods are illustrated in Figure 9. One can see that the SH-based methods adhered better to the contours of regions than the SLIC method. In particular, compared with others, the SH+FDAG and SH+AAGK methods retained more contours of regions in the superpixel segmentation. It is believed that some subsequent tasks (e.g., remote sensing imagery segmentation and classification [46,47]) will benefit from accurately segmented superpixels.

In order to see whether or not the quantitative evaluation results of our method were significantly different from those of other methods, we selected the cases corresponding to 300, 700, 1100, 1500, and 1900 superpixels, respectively. For each case, we also employed the non-parametric Friedman test with the null hypothesis that all the methods obtained the same results. The *p*-value results of the Friedman test are presented in Table 4. As indicated by these results, the null hypothesis was rejected in each case. That is, the average ASA and average UE values obtained by different methods had significant differences in each case. Then, in the post-hoc Nemenyi tests, we selected our method as the control method. According to Equation (30), the critical distance in this experiment was 0.8430. The average rank of each method in each case is reported in Table 5 and Table 6. As can be seen, our method performed significantly different from the SLIC, SH+SFE, and SH+SCO methods in most scenarios, while achieving a competitive performance compared with the SH+AAGK method.

### 5.5. Evaluation on the NSEMS Dataset

**Figure 10:**
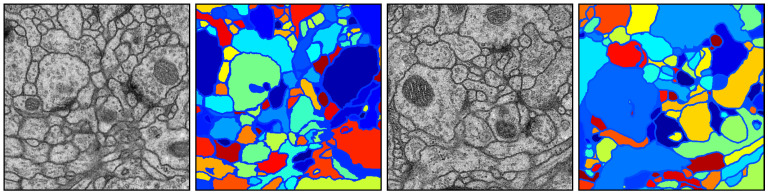
Sample images as well as their GT segmentation maps taken from the NSEMS dataset. **First and third columns:** Original images. **Second and fourth columns:** Corresponding GT segmentation maps.

The NSEMS dataset comprises 30 grayscale electron microscopy images, each of which has a resolution of 512×512, as well as the GT segmentation maps. Two sample images and the corresponding GT segmentation maps are illustrated in Figure 10. Note that there is one GT segmentation map for each microscopy image. In this experiment, we did not test the SH+SED method since it is not applicable to grayscale images [27].

**Figure 11:**
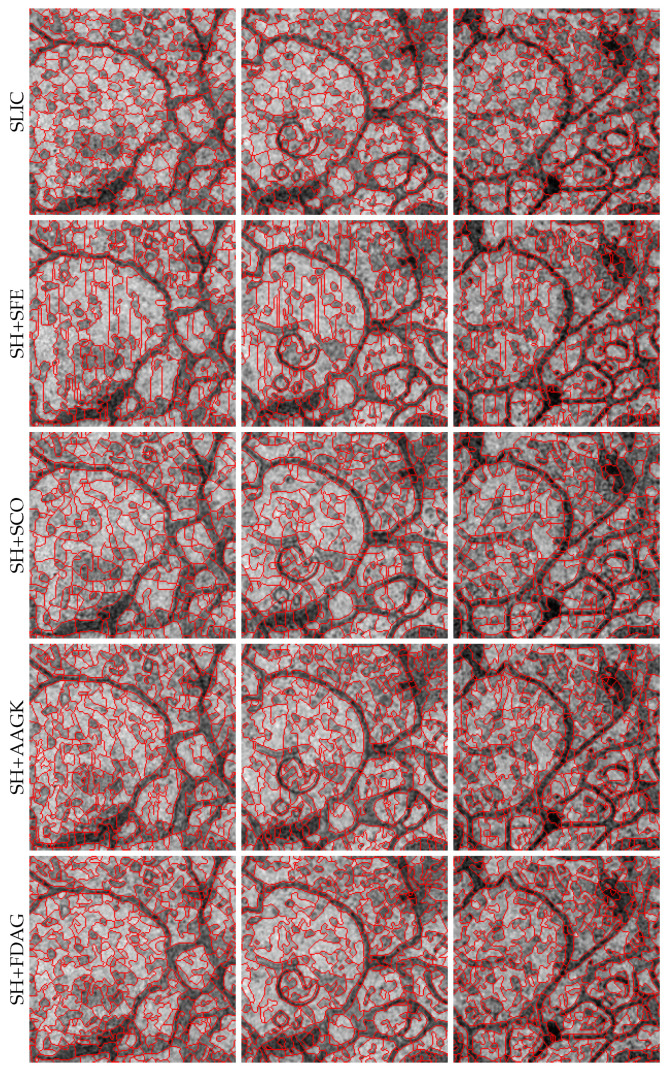
Superpixel maps yielded by different methods on sample images taken from the NSEMS dataset. The number of superpixels in each full superpixel map was set as 2000. For a better visualization, zoomed-in versions are displayed.

For each method, the curves of average ASA and average UE values over all the 30 images with respect to different numbers of superpixels are shown in Figure 5e,f. It can be seen that our method performed better than all the other methods, achieving higher average ASA values and lower average UE values than the competing methods in most cases. Note that the SH+SFE method did not obtain the best performance in terms of the average ASA and average UE, which is similar to the experimental results obtained on the SBAIS dataset. This is because the edge detection model in the SFE method was trained on the BSDS500 dataset, and as such, it underperformed on electron microscopy images. Among all the methods, the SH+SCO method performed the worst. In addition, the SLIC method performed better for fine superpixel segmentation than for coarse superpixel segmentation, which is consistent with the previously reported experimental results. For illustration, Figure 11 displays the superpixel maps yielded by different methods. It can be observed that our method adhered to the contours better than the competing methods.

To see whether or not the evaluation results obtained by our method were significantly different from those of other methods, we selected the cases corresponding to 4000, 6000, 8000, 10,000, and 12,000 superpixels, respectively. For each case, we also applied the non-parametric Friedman test with the null hypothesis that all the methods obtained the same evaluation results in terms of the average ASA and average UE, respectively. We list the *p*-value results of the Friedman test in Table 7. As indicated by these results, the null hypothesis was rejected in each case. That is, the average ASA and average UE values obtained by different methods had significant differences in each case. In the post-hoc Nemenyi test, we selected our method as the control method, since it obtained the best average ASA and average UE in all the selected cases. Using Equation (30), we computed the critical distance in this experiment as 1.1137. According to the average rank values of all the methods reported in Table 8 and Table 9, our method performed significantly differently from the competing methods in most scenarios.

Furthermore, from the experimental results obtained on the three datasets, it can be seen that the proposed SH+FDAG method achieved a more stable performance than the competing methods. This illustrates that our method is less dataset-dependent than the competing methods.

### 5.6. Application to Saliency Detection

We confirmed that our method has an overall advantage in superpixel segmentation compared with the competing methods. In the following we show how the superpixels yielded by our method facilitate subsequent processing with an example task of saliency detection. In computer vision, saliency detection is aimed at finding the pixels that belong to the most salient region attracting the attention of human visual system. In the literature, there are quite a few saliency-detection methods that are built on superpixel maps [2]. A prominent method was proposed by Qin et al. [48], in which the superpixels evolve into a saliency detection result based on a cellular automaton mechanism (SCA method). In the SCA method, the superpixels in the vicinity of the image frame are grouped into several background clusters. Subsequently, a propagation mechanism based on a cellular automaton is designed to exploit the intrinsic dissimilarity between each superpixel and the background clusters in terms of color difference and spatial distance. The saliency map is obtained after a number of iterations. In the original method, the superpixels used are generated by the SLIC method (SCA+SLIC) [48].

**Figure 12:**
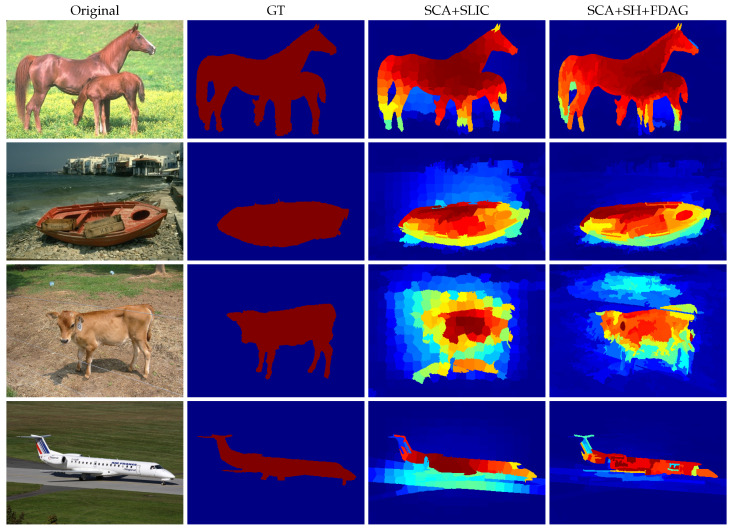
Sample results of saliency detection obtained by the SCA+SLIC and SCA+SH+FDAG methods. SCA:saliency detection based on cellular automaton.

In this experiment, we used the SCA method based on superpixels generated by our method (SCA+SH+FDAG) to obtain the saliency detection results on four sample images taken from the extended complex scene saliency dataset (http://www.cse.cuhk.edu.hk/~leojia/projects/hsaliency/dataset.html) [49]. We also compared our method with the original SCA+SLIC method. The parameters were configured according to the original implementation [48]. In particular, the number of superpixels in each method was set to 300. Figure 12 illustrates the saliency detection results. Compared with the SCA+SLIC method, the SCA+SH+FDAG method yielded saliency maps with a higher accuracy. For instance, in the second row of Figure 12, the salient boat was well-separated from the non-salient regions by the SCA+SH+FDAG method, while the SCA+SLIC method also assigned significant saliency values to some non-salient pixels. Complementary to the visual comparison, we also quantitatively evaluated the saliency detection performance of the SCA+SLIC and SCA+SH+FDAG methods in terms of the mean absolute error (MAE) representing the difference between the obtained saliency map and the GT saliency map [2]:

(31) $$\mathrm{MAE}=\frac{1}{N_{J}}\sum_{i=1}^{N_{J}}\left|J_{\mathrm{sl}}\left(m_{i}\right)-J_{\mathrm{gt}}\left(m_{i}\right)\right|\;,$$

where $m_{i}$ denotes the pixel location, $J_{\mathrm{sl}}$ and $J_{\mathrm{gt}}$ represent the obtained saliency map and the GT saliency map, respectively, $N_{J}$ stands for the number of pixels in the saliency map, and |·| denotes the absolute value of a real number. For saliency detection, a lower MAE value is preferred. The evaluation results obtained on the sample images are presented in Table 10. As can be seen, our method obtained lower MAE values on all the sample images, outperforming the SCA+SLIC method.

## 6. Conclusions

In this paper, aiming at accurate superpixel segmentation, we elaborated a method to measure the edge strength using FDAG kernels. In addition, we introduced the SH method, which takes the edge strength into consideration in generating superpixels. We also presented an SH-based superpixel segmentation method that incorporates the anisotropic edge strength. Experimental results validated that, in the SH-based superpixel segmentation, the proposed FDAG-based edge strength measurement method has advantages over the competing methods, including the SFE method [28], the SCO method [26], the AAGK method [19], and the SED method [27]. In particular, compared with the original SH method [9] in which the edge strength is provided by the learning-based SFE method [28], our method is less dataset-dependent, facilitating the SH-based superpixel segmentation of different kinds of images. Furthermore, we also illustrated that the superpixels yielded by our method can facilitate a subsequent saliency detection task.

## Figures and Tables

**Table 1:** *P*-value of the Friedman test of the average ASA and average UE in selected cases obtained on the BSDS500 dataset.

Item	Number of Superpixels				
	100	300	500	700	900
average ASA	1.7221$\;\times \;10^{-46}$	7.0197$\;\times \;10^{-47}$	1.1490$\;\times \;10^{-64}$	1.4271$\;\times \;10^{-61}$	6.1600$\;\times \;10^{-65}$
average UE	4.6868$\;\times \;10^{-56}$	1.6198$\;\times \;10^{-56}$	4.2295$\;\times \;10^{-66}$	1.1916$\;\times \;10^{-61}$	6.8556$\;\times \;10^{-64}$

**Table 2:** The average ASA rank of each method in selected cases obtained on the BSDS500 dataset. For ASA evaluation, a higher average rank value is preferred.

Methods	Number of Superpixels				
	100	300	500	700	900
SLIC	1.8500 *	2.1200 *	2.7500 *	3.2575 *	3.4550 *
SH+SFE	4.2050	4.4425	4.3200 *	4.6550	4.6750
SH+SCO	3.5172 *	3.6850 *	2.9525 *	3.1350 *	3.1800 *
SH+AAGK	3.8800	3.8300 *	3.6375 *	3.2300 *	3.5300 *
SH+SED	3.3200 *	2.7475 *	2.2925 *	2.0350 *	1.7400 *
SH+FDAG ${}^{†}$	4.2275	4.1750	5.0475	4.6875	4.4200

The symbol ${}^{†}$ denotes the control method. The symbol * indicates that the value is significantly different from the value of the control method.

**Table 3:** The average UE rank of each method in selected cases obtained on the BSDS500 dataset. For UE evaluation, a lower value of average rank is preferred.

Methods	Number of Superpixels				
	100	300	500	700	900
SLIC	5.3700 *	5.1850 *	4.7325 *	4.1650 *	4.0450 *
SH+SFE	2.7850	2.3900	2.4400	2.2700	2.0600 *
SH+SCO	3.3500 *	3.3200 *	3.8975 *	3.7350 *	3.7650 *
SH+AAGK	3.2900 *	3.3700 *	3.6150 *	3.9400 *	3.5950 *
SH+SED	3.5000 *	3.9100 *	4.2700 *	4.6600 *	4.9400 *
SH+FDAG ${}^{†}$	2.7050	2.8250	2.0450	2.2300	2.5950

The symbol ${}^{†}$ denotes the control method. The symbol * indicates that the value is significantly different from the value of the control method.

**Table 4:** *P*-value of the Friedman test of the average ASA and average UE in selected cases obtained on the SBAIS dataset.

Item	Number of Superpixels				
	300	700	1100	1500	1900
average ASA	1.2926$\;\times \;10^{-28}$	2.7848$\;\times \;10^{-30}$	1.9566$\;\times \;10^{-35}$	7.4836$\;\times \;10^{-40}$	8.2617$\;\times \;10^{-47}$
average UE	3.3323$\;\times \;10^{-34}$	1.0321E $\;\times \;10^{-38}$	5.1945$\;\times \;10^{-43}$	1.4668$\;\times \;10^{-44}$	1.8858$\;\times \;10^{-50}$

**Table 5:** The average ASA rank of each method in selected cases obtained on the SBAIS dataset. For ASA evaluation, a higher value of average rank is preferred.

Methods	Number of Superpixels				
	300	700	1100	1500	1900
SLIC	2.1625 *	3.0750 *	3.6938 *	4.2500	4.5125
SH+SFE	4.3875	3.8875 *	3.7875 *	3.4875 *	3.2125 *
SH+SCO	2.9750 *	2.7250 *	2.5375 *	2.5375 *	2.5375 *
SH+AAGK	4.3750	4.4125	4.4250	4.3375	4.0750 *
SH+SED	2.4250 *	1.9125 *	1.6125 *	1.4500 *	1.4000 *
SH+FDAG ${}^{†}$	4.6750	4.9875	4.9438	4.9375	5.2625

The symbol ${}^{†}$ denotes the control method. The symbol * indicates that the value is significantly different from the value of the control method.

**Table 6:** The average UE rank of each method in selected cases obtained on the SBAIS dataset. For UE evaluation, a lower value of average rank is preferred.

Methods	Number of Superpixels				
	300	700	1100	1500	1900
SLIC	5.0375 *	4.3188 *	3.6500 *	3.1250 *	2.7625 *
SH+SFE	2.3500	2.8000 *	2.8750 *	3.1375 *	3.3300 *
SH+SCO	4.2063 *	4.5563 *	4.7250 *	4.7125 *	4.8125 *
SH+AAGK	2.5750	2.5875	2.5875	2.6750 *	2.9125 *
SH+FDAG ${}^{†}$	2.3563	1.7750	1.8000	1.8125	1.6125

The symbol ${}^{†}$ denotes the control method. The symbol * indicates that the value is significantly different from the value of the control method.

**Table 7:** *P*-value of the Friedman test of the average ASA and average UE in selected cases obtained on the NSEMS dataset.

Item	Number of Superpixels				
	4000	6000	8000	10,000	12,000
average ASA	2.9556$\;\times \;10^{-18}$	8.4506$\;\times \;10^{-19}$	1.9923$\;\times \;10^{-20}$	5.7408$\;\times \;10^{-20}$	1.7971$\;\times \;10^{-21}$
average UE	7.6737$\;\times \;10^{-17}$	1.0475$\;\times \;10^{-15}$	3.6067$\;\times \;10^{-17}$	5.3294$\;\times \;10^{-16}$	2.5874$\;\times \;10^{-20}$

**Table 8:** The average ASA rank of each method in selected cases obtained on the NSEMS dataset. For ASA evaluation, a higher value of average rank is preferred.

Methods	Number of Superpixels				
	4000	6000	8000	10,000	12,000
SLIC	2.0667 *	2.9000 *	3.5333 *	3.3667 *	4.0333
SH+SFE	3.8000	3.4000 *	3.0000 *	2.9667 *	2.6333 *
SH+SCO	1.5332 *	1.2333 *	1.1333 *	1.0667 *	1.0667 *
SH+AAGK	2.7000 *	2.4667 *	2.3333 *	2.6000 *	2.4333 *
SH+FDAG ${}^{†}$	4.9000	5.0000	5.0000	5.0000	4.8333

The symbol ${}^{†}$ denotes the control method. The symbol * indicates that the value is significantly different from the value of the control method.

**Table 9:** The average UE rank of each method in selected cases obtained on the NSEMS dataset. For UE evaluation, a lower value of average rank is preferred.

Methods	Number of Superpixels				
	4000	6000	8000	10,000	12,000
SLIC	4.1333 *	3.5667 *	2.6333 *	2.9000 *	1.9667
SH+SFE	2.2000	2.5667 *	3.1000 *	3.1667 *	3.4667 *
SH+SCO	4.1000 *	4.1667 *	4.4667 *	4.5000 *	4.7667 *
SH+AAGK	3.4333 *	3.7000 *	3.8000 *	3.4333 *	3.6333 *
SH+FDAG ${}^{†}$	1.1333	1.0000	1.0000	1.0000	1.1667

The symbol ${}^{†}$ denotes the control method. The symbol * indicates that the value is significantly different from the value of the control method.

**Table 10:** Saliency detection evaluation results in terms of mean absolute error (MAE) obtained on the sample images.

Methods	Index of the Sample Images			
	1	2	3	4
SCA+SLIC	0.0710	0.1094	0.1921	0.0789
SCA+SH+FDAG	0.0585	0.0846	0.1561	0.0519
